# The Temporal Spectrum of Adult Mosquito Population Fluctuations: Conceptual and Modeling Implications

**DOI:** 10.1371/journal.pone.0114301

**Published:** 2014-12-05

**Authors:** Yun Jian, Sonia Silvestri, Jeff Brown, Rick Hickman, Marco Marani

**Affiliations:** 1 Nicholas School of the Environment, Duke University, Durham, North Carolina, 27708, United States of America; 2 Mosquito Control Department, Brunswick County Government, Brunswick, North Carolina, 28422, United States of America; 3 Department of Civil and Environmental Engineering, Duke University, Durham, North Carolina, 27708, United States of America; 4 Department of Civil, Architectural, and Environmental Engineering, University of Padova, Padova, Italy; National Taiwan University, Taiwan

## Abstract

An improved understanding of mosquito population dynamics under natural environmental forcing requires adequate field observations spanning the full range of temporal scales over which mosquito abundance fluctuates in natural conditions. Here we analyze a 9-year daily time series of uninterrupted observations of adult mosquito abundance for multiple mosquito species in North Carolina to identify characteristic scales of temporal variability, the processes generating them, and the representativeness of observations at different sampling resolutions. We focus in particular on *Aedes vexans* and *Culiseta melanura* and, using a combination of spectral analysis and modeling, we find significant population fluctuations with characteristic periodicity between 2 days and several years. Population dynamical modelling suggests that the observed fast fluctuations scales (2 days-weeks) are importantly affected by a varying mosquito activity in response to rapid changes in meteorological conditions, a process neglected in most representations of mosquito population dynamics. We further suggest that the range of time scales over which adult mosquito population variability takes place can be divided into three main parts. At small time scales (indicatively 2 days-1 month) observed population fluctuations are mainly driven by behavioral responses to rapid changes in weather conditions. At intermediate scales (1 to several month) environmentally-forced fluctuations in generation times, mortality rates, and density dependence determine the population characteristic response times. At longer scales (annual to multi-annual) mosquito populations follow seasonal and inter-annual environmental changes. We conclude that observations of adult mosquito populations should be based on a sub-weekly sampling frequency and that predictive models of mosquito abundance must include behavioral dynamics to separate the effects of a varying mosquito activity from actual changes in the abundance of the underlying population.

## Introduction

A detailed understanding of mosquito population dynamics under natural environmental forcing requires the observation and understanding of ecological processes over a wide range of time scales. For example, the effect of rainfall on mosquito oviposition has been documented to be dependent on the time scale: the effect is negative over short and intermediate time scales, due to diluted nutrients, reduced mosquito activity, and egg removal, and it is positive over long time scales because of increased habitat extent and relative humidity [1]. Adult mosquito activity (such as host seeking) can also respond quickly to meteorological forcings [2], [3], thus inducing fast response times of apparent population abundance. Many mosquito species have relatively short generation times. Laboratory experiments show that, under favorable conditions, less than 3 weeks may be required between two successive generations of *Ae.vexans* [2]. Mosquito oviposition and feeding processes occur at hourly and daily scales [1], [4], [5], and endogenous and exogenous driving factors typically vary on weekly to monthly scales [6]-[12].

The sampling resolution should of course adequately cover such a wide range of scales, but few studies address the role of temporal resolution in mosquito sampling [10], [13]-[16], which is most commonly carried out over one single night with a weekly frequency [6], [8], [11], [17]-[21]. Daily, long-term studies of adult mosquito populations in their environment are, in fact, the exception rather than the rule. Among the exceptions is the work by Shaman et al. [22], who focus on linking water availability and mosquito abundance at the monthly scale. Daily data are also used in Chuang et al [23], who aggregate them into weekly means to explore the merit of using satellite and in situ weather measurements as drivers for mosquito modeling. Both these contributions take advantage of the increased reliability of weekly/monthly abundance estimates afforded by averaging daily data, but do not explore the full extent of the temporal scales covered by a daily dataset.

Indeed, particularly in the case of adult mosquito populations in natural conditions, an important question concerns the choice of the sampling frequency which can capture the governing population dynamical mechanisms. In fact, the degree with which observations represent the population being studied is a general and fundamental problem that should always be explicitly addressed in ecological studies [24]–[26]. In the present case, the representativeness of observations of adult mosquito abundance with respect to the underlying population dynamics depends crucially on the time scales over which the size of the population changes, and on the factors that may affect the relation between observations and the actual abundance.

In this framework, the aims of this paper are the identification of the range of temporal scales over which mosquito population variability occurs, the attribution of these temporal scales of fluctuation to the exogenous and endogenous dynamical mechanism generating them, and the inference of implications for observational requirements and modelling approaches. To this end we analyze, through Fourier Transform and mechanistic population models, a unique 9-year time series of uninterrupted daily abundance observations in North Carolina involving a large number of mosquito species, produced and maintained by the Mosquito Control Department in Bolivia (NC).

## Methods

### Study area and data

**Figure 1:**
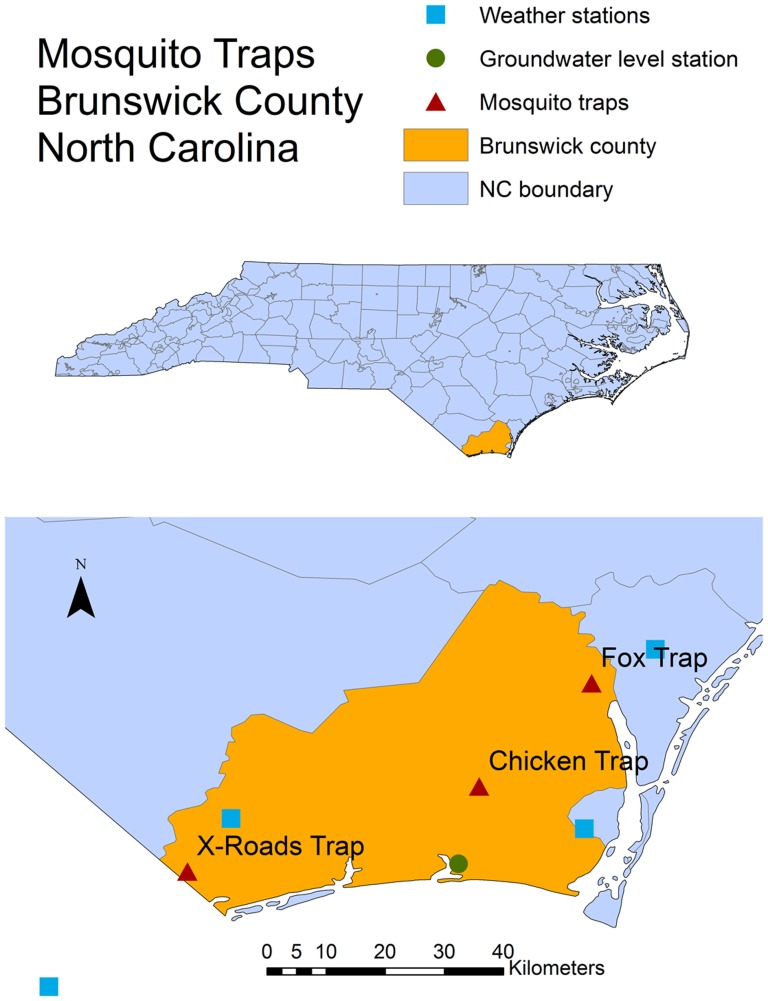
Study area, sampling sites and weather stations.

The Mosquito Control Department in Brunswick County (North Carolina – USA) was established to monitor and prevent mosquito-borne diseases, with particular reference to Eastern Equine Encephalitis (EEE) and West Nile Virus (WNV), among the most severe mosquito-borne diseases in temperate semi-humid areas. The ornithophillic *Culiseta melanura*, and several other species, including *Aedes vexans*, have been implicated as mammalian bridge vectors of EEE, as well as of other arboviruses such as WNV [27]–[30]. Since 2004, the Mosquito Control Department of Brunswick County has been routinely collecting daily abundance of adult mosquito at three locations using New Jersey Light Traps (NJLT, Figure 1) all year around. NJLT's were placed 4 feet off the ground at chest height. The trap light source is a 25 watt frosted incandescent light bulb. All traps were hard wired and plugged into a 120 V all-weather outlet. The light source and fan were run continuously. Trap collections are made every morning between 8 and 10 am, 7 days a week. The mosquito sampling was carried out on the Brunswick county government property (the “Chicken Trap”, 34.059°, −78.168°, contact Jeffrey Brown Jbrown@brunsco.net for future permissions) as well as on private property (the “Fox trap”, 34.216°, −78.000°, and “X-roads trap”, 33.930°, −78.611°, for future permissions contact Jeffrey Brown Jbrown@brunsco.net and Rick Hickman rickhickman@atmc.net). Field studies did not involve endangered or protected species.

We focus here on the “Chicken Trap” site (close to several woodland pools and to a sentinel chicken site providing blood bait) because of the comparatively large sample size and because expert opinions from the Brunswick County Mosquito Control Department suggest that the population dynamic recorded at the "Chicken trap" sentinel site is well-aligned with changes in mosquito population abundance over large areas of the County. Daily weather data (temperature, precipitation, dew point, and wind speed) at the closest National Climate Data Center (NCDC) station were acquired (Station USW00013748, about 34 km from the “Chicken Trap” site, http://gis.ncdc.noaa.gov, http://gis.ncdc.noaa.gov). Ground water level observations were also downloaded from the U.S. Geological Survey real-time groundwater level network (Station 335629078115407, 13 km from the “Chicken Trap” site http://groundwaterwatch.usgs.gov/). These data were used to study the role of exogenous environmental controls as drivers of mosquito population dynamics.

*Cs.melanura* is identified as the primary vector of EEE disease. This mosquito species feed primarily on birds and lay eggs in underground crypts. It is a multivoltine species with an exceptionally low development rate and a tolerance to cold weather. It can overwinter in multiple larval stages [27], [29], [31]–[33]. *Ae.vexans* is a floodwater mosquito species ubiquitously found in the USA. It is important because of its abundance, widespread distribution, and role as the vector of multiple diseases. This species lays eggs in soils subject to transient flooding. The eggs hatch and complete their development when submerged under water. Unlike *Cs.melanura*, *Ae.vexans* overwinters as eggs [2], [34], [35].

### Data analysis

Various time series analysis approaches have been developed and applied in the context of population ecology (e.g. phase space models, autoregressive models, and Bayesian models [24], [36]–[44]). The objectives of this work require the identification and analysis of the characteristic scales of fluctuation of mosquito abundance. We tackled this problem by first applying Fourier analysis to detect and identify, in the observed adult mosquito abundance and environmental forcing time series, characteristic fluctuation time scales. We then experimented by artificially degrading the data resolution to the weekly scale to observe how fast scales of fluctuation and inferred statistical properties changed with observation frequency. Finally, we used an Individual Based Simulation model (IBS model), as well as density-dependent population models, to comparatively explore the relative importance of mosquito activity and of endogenous and exogenous controls in determining the observed scales of fluctuation and the overall population dynamics.

#### Fourier Analysis

Discrete Fourier Transform (DFT) decomposes a time series into the sum of sinusoidal functions with varying periods T_i_ =  (N ⋅Δt)/i (i = 0, …, N/2) [45] (where N is the sample size and Δt is the sampling interval). This representation is very useful to detect seemingly irregular hidden periodicities. DFT provides, as a result of the analysis, the amplitude of the fluctuations corresponding to each discrete period T_i_, hence allowing identification of possible dominant periodicities (i.e. having relatively large amplitudes). Other analysis methods, such as wavelet analysis, provide further sophistication, e.g. in analyzing non-stationary time series [46]. However, the focus here is the identification of characteristic periodicities in adult mosquito populations (which may e.g. arise due to the presence of characteristic time scales in environmental forcings and/or in mosquito life cycles), and the DFT (employed in previous population ecology studies, e.g. see [46]–[48]) is an efficient tool that suffices this objective. One important notion related to the DFT is the Nyquist theorem, which establishes that the shortest periodicity (i.e. the fastest dynamics) that can be captured when a process is sampled at a resolution Δt is equal to 2Δt (corresponding to the Nyquist frequency, 1/(2Δt)). In the case of mosquito abundance observations, when adult mosquitoes are collected once per week, the shortest periodicity that can be resolved is 2 weeks: fluctuations occurring over shorter time scales will remain undetected in the data and will be seen as “noise”. The dataset used here has a resolution of 1 day, such that the shortest periodicity that can be resolved is equal to 2 days: this dataset allows us to explore, in the field and under natural conditions, the full range of periodicities that may be present in population dynamics of adult mosquitoes. As customary, we represent results from the DFT through the power spectrum, S(T), which represents the square of the oscillation amplitude of the sinusoidal component of period T in the Fourier decomposition of the time series studied.

### Autocorrelation, partial correlation, and data resampling

We resampled the daily observation time series at a 7-day frequency to evaluate how a degraded temporal resolution affects the temporal scales of fluctuation captured by the time series. We obtained 7 subsampled time series, each “collected” on a different day of the week. We computed the AutoCorrelation Function (ACF) and the Partial AutoCorrelation Function (PACF) for the original dataset and for the sub-sampled ones. The k-th element of the ACF provides a measure of the average correlation between the abundance values *x_t_*, and *x_t-k_*, k time steps apart. The PACF is obtained by removing from the correlation between *x_t_* and *x_t-k_* the indirect correlation associated with the intermediate terms *x_t-1_*, *x_t-2_*, …, *x_t-k+1_*, such that only the direct correlation between *x_t_*, and *x_t-k_* is retained [43].

#### Models

**Figure 2:**
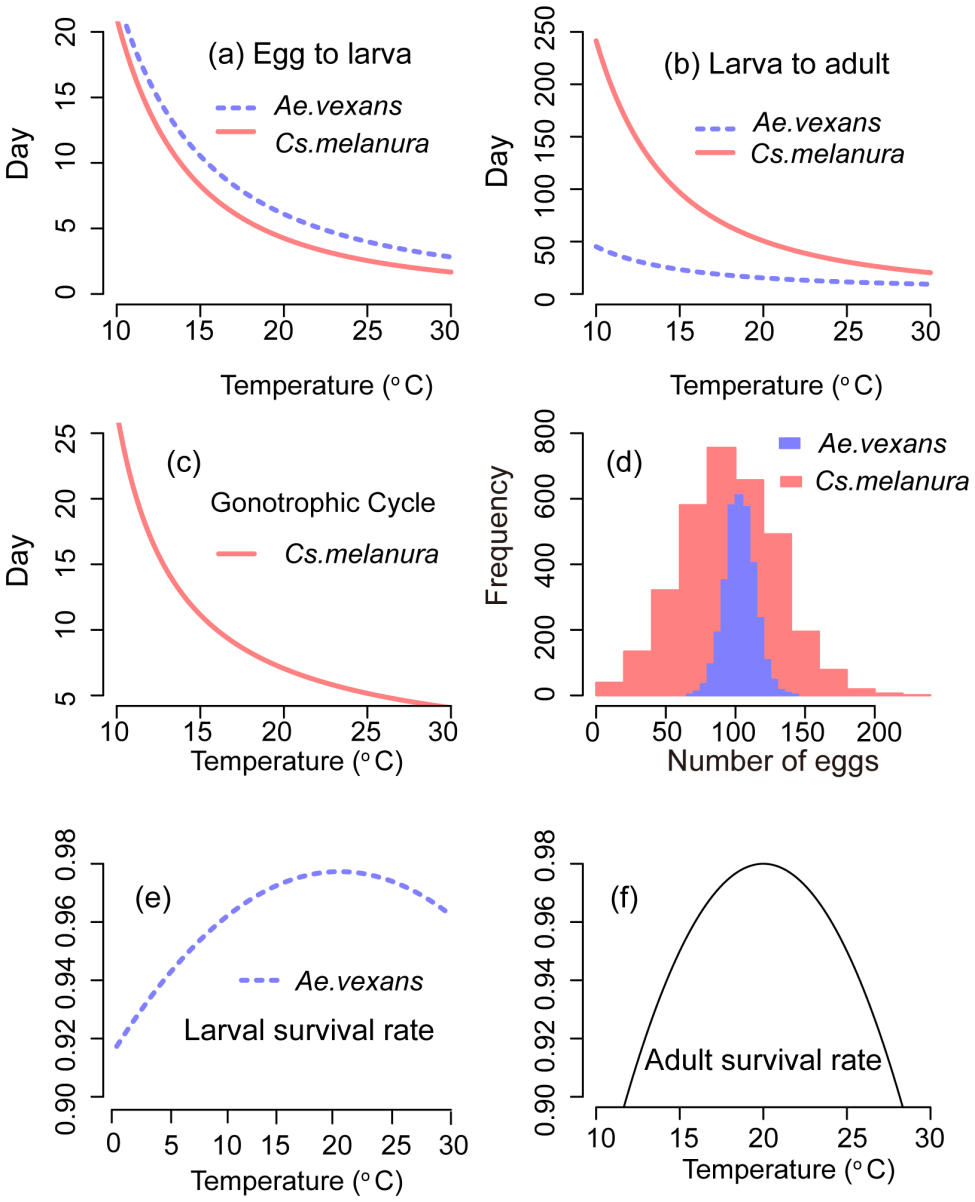
Main biological parameters as assumed in the IBS model. (a) Transition time from egg to larva as a function of temperature. (b) Transition time from larva to adult as a function of temperature. (c) Length of gonotrophic cycle for *Cs.melanura* as a function of temperature. (d) Marginal distribution of the number of eggs laid per batch. (e) Larval survival rate as a function of temperature for *Ae.vexans*. (f) Adult survival rate as a function of temperature for both *Cs.melanura* and *Ae.vexans*.

With the aim of identifying the statistical properties in the observed abundance of adult mosquitoes associated with distinct population dynamical mechanisms, we constructed Individual-Based life cycle Simulation (IBS) models for Ae.vexans and Cs.melanura populations. The IBS models explicitly describe three mosquito life stages: egg, larva/pupa, and adult [2]. Each stage is characterized by a distribution of the time spent in that stage, dependent on physiology, environmental conditions, and population density (Figure 2 and Figure S1 in File S1). Each stage is characterized by a survival rate, also a function of environmental conditions (Figure 2). We based our description of the distributions of the time spent in each stage and of the survival rate for each stage on existing literature, to minimize the number of parameters which require ad hoc assumptions (Figure 2). No calibration of the models was performed as the objective is here to obtain a realistic representation of adult mosquito abundance fluctuations, rather than the numerical reproduction of a specific sample.

The time spent in each life stage by each individual is drawn from a normal distribution whose mean depends on the temperature averaged over a 10-day window (to represent temperature conditions throughout the developing period), and a fixed standard deviation (1 day). The mean “residence” time (*d*) in each stage is assumed to decrease with the average temperature according to a power law (Figure 2): *d =  A ⋅ T ^−a^* [2]. The literature-derived exponents adopted are as follows: a = 1.90 for *Ae.vexans* embryogenesis, a = 1.86 for *Ae.vexans* larval/pupal stage, a = .30 for *Cs.melanura* embryogenesis, and a = 2.25 for *Cs.melanura* larval/pupal stage [2], [29], [35], [49]. The gonotrophic cycle in adult mosquitoes includes copulation, blood search, blood digestion, and egg development in the female body. The length of the gonotrophic cycle for *Cs.melanura* is assumed to be drawn from a Gaussian distribution with a mean which decreases with increasing temperature and a unit standard deviation [32]. The length of the gonotrophic cycle for *Ae.vexans* is assumed to be a truncated normal distribution with a mean of 10 days, standard deviation of 1 day, upper bound of 13 days and lower bound of 7 days [2], [35] (Figure 2 and File S2). The survival rates for the larval/pupal and adult stages of *Ae.vexans*, and the adult stage of *Cs.melanura* are quadratic functions of the 10-day moving average temperature (Figure 2) [32], [35], [49]. The larval/pupal survival rate of *Cs.melanura* is assumed to be 1 when the current larval abundance is zero and 10-day moving average of precipitation is larger than 1 mm/d as there is no information for this species. We further assume that the survival rates of larvae and adults for both species decreases when the 10-day moving average of precipitation is below 1 mm/day to mimic a documented sensitivity of survival rates on moisture and water availability [3], [17]. Finally, survival rates for egg and larval stages for both species are assumed to decrease linearly with abundance to reflect documented density dependence effects [6] (File S2).

Under natural conditions only a fraction of female mosquitoes is successful in obtaining a blood meal and are able to oviposit. The probability of success is represented by a Bernoulli distribution with a mean that linearly increases with the 10-day moving average of relative humidity for *Ae.vexans*, and a mean which is an increasing quadratic function of the 10-day moving average of temperature for *Cs.melanura* (File S2) [2], [32]. After oviposition, engorged females can start another gonotrophic cycle until they die. This model representation realistically reproduces (under current weather conditions) the fact that the large majority of females only have the opportunity of one blood meal and oviposit only once during their lifetime [35]. Only a small proportion of females succeeds in obtaining a second blood meal and oviposit a 2nd batch of eggs. The number of eggs laid by each adult per batch is assumed to have a normal distribution, with a mean that increases with precipitation amount, as water is more likely available for oviposition (Figure 2).

Details on the development rate and the values of the controlling factors for each development stage can be found in File S2. Such values were obtained from the wide relevant literature, as noted above, and we purposely avoided ad hoc calibrations because the objective is to identify the signature of each individual developmental process on the resulting population variability, rather than to maximize the ability of the model to reproduce a specific observed time series. The models (i.e. defined by one set of parameter values for each species) were run under three configurations: (1) observed environmental forcings (temperature and rainfall in 2004-2012) (2) observed temperature forcing only (effect of rainfall on survival rate, oviposition and egg hatching “ turned off ”) (3) endogenous dynamics only (fixed temperature -T = 18°C - and rainfall effect turned off). The three model configurations allow us to separate and identify the population fluctuations and periodicities caused by endogenous and environmental controls.

The IBS models produce, at each time step, the abundance of the “actual” adult mosquito population (the abundances of the other life stages, also computed at each time step, are not analyzed here due to a lack of the corresponding observations). We further assumed that the observed population, i.e. the number of adults that would be attracted and captured by a trap, is a varying fraction of the underlying actual adult population. The dependence of this “activity fraction” on rainfall was estimated based on observations as follows. We first calculated the ratio of the current abundance to the maximum abundance in a moving window of 31 days. The rationale for this estimate is that the maximum abundance over a relatively homogeneous period should be a good proxy for the fraction of the underlying population that can be captured under the most favorable environmental conditions. The median of the activity ratio computed over discrete intervals of rainfall intensity was then plotted against current day rainfall. This analysis indicates that the activity ratio increases with rainfall at low intensities and decreases steeply for rainfall intensities above a threshold (Figure S2 in File S1). This nonlinear dependence of the activity ratio on rainfall was used to represent rainfall-activity effects in the IBS model.

We note here that, while the IBS model formulation indeed includes several assumptions, we have extensively experimented with a wide variety of such assumptions and parameter values, and found the impact on the emerging dynamical time scales, and particularly on the fastest and slowest time scales of population fluctuations, to be limited. Because of the stochastic nature of the IBS models, we ran the models 20 times for each species, to obtain ensemble means as a basis for further discussion.

Two classic and commonly used population models were applied to the daily abundance observations of *Ae.vexans* and *Cs.melanura*: the Ricker model and the Gompertz-logistic model [7], [24] (Table 1). These models are defined through specific relations between per-capita growth rate and abundance: the relation is linear in the Ricker model and log-linear in the Gompertz formulation. Through the application of these models we tested for the existence of density dependence with lag between 0 and 5 days, to explore the possible effects of delayed density control. We also compared the statistical properties of the population dynamics generated by these models with those from observations, to identify which features can indeed be reproduced using canonical population modeling. Environmental forcings were included in the models as additive terms, to account for the dependence of the carrying capacity and the maximum per-capita growth rate on environmental conditions [6], [8]. These models were calibrated (in R 2.15.1) as linear models using the least squares method (see File S3 for estimated parameter values). It is known that least square methods applied to ecological models can lead to improper estimates of parameter values [50]. However, the focus of our analyses is not the identification of “true” parameter values, but the evaluation of the ability of density-dependent population model formulations to capture observed correlation structures. We will thus focus on comparing modeled and observed correlation structures rather than on quantifying bias in parameter estimates. The calibrated density-dependent models were used to generate synthetic time series, and their ACF and PACF were compared with those of the original observations and of the time series produced using the IBS model.

**Table 1:** Density-dependent models (showing lag 0 as examples).

Model name	Recursive form
Ricker model	*N_t+1_* = *N_t_* × exp(*r_m_* (1-*N_t_/K*))
Gompertz-logistic model	*N_t+1_* = *N_t_* × exp(*r_m_* (1-log*N_t_/*log*K*))

Where t is index of sample date; *N* is adult mosquito abundance; *r_m_* is the maximum per capital growth rate; *K* is the carrying capacity.

## Results

**Figure 3:**
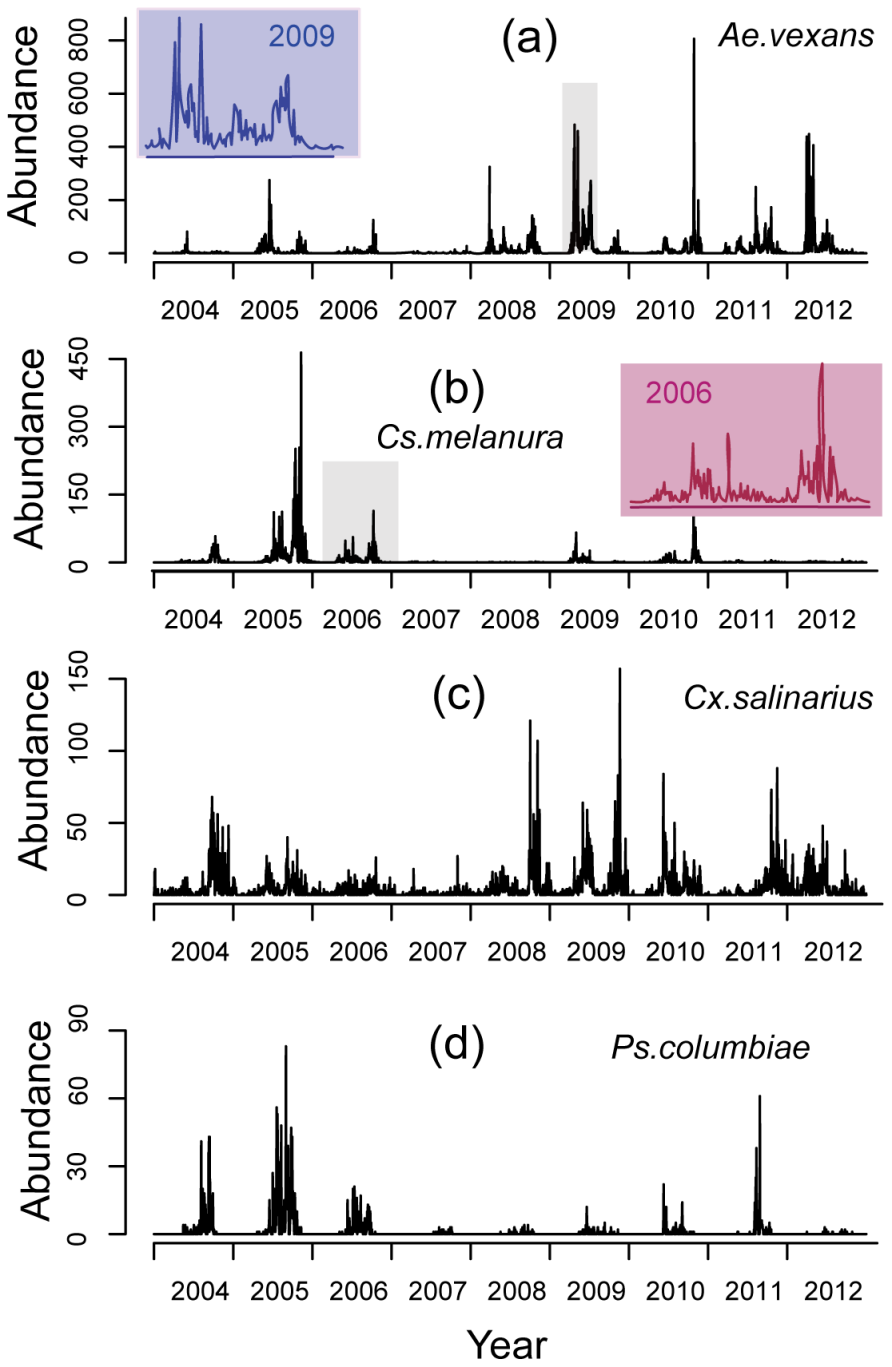
Observed abundance for the 4 dominant species at the Chicken Trap site in the period 2004 – 2012. The subpanels show details of the abundance fluctuations for two specific periods identified by the grey windows in (a) and (b).

We analyze the observed statistical properties of the four most abundant species in the studied area: *Aedes vexans*, *Culex salinarius*, *Culiseta melanura*, and *Psorophora columbiae* (Figure 3). *Cs.melanura* and *Ae.vexans* are connected with EEE and WNV diseases, hence the additional interest and further analyses reported later. Figure 3 shows large and very rapid fluctuations in the daily abundance of adult mosquitoes: for example, more than 800 individuals of *Ae.vexans* were collected on Oct-27-2010, while only about 50 individuals were collected 2 days before and after. This dramatic change in the abundance is much more rapid than would be allowed by physiologically possible generation times (about 3 weeks under favorable conditions [2]). Indeed, large differences (several order of magnitudes in 1–2 days during the growing season) are present throughout the 9-year dataset (Figure 3), which are likely attributable to the changing proportion of active individuals rather than to changes in the actual population.

**Figure 4:**
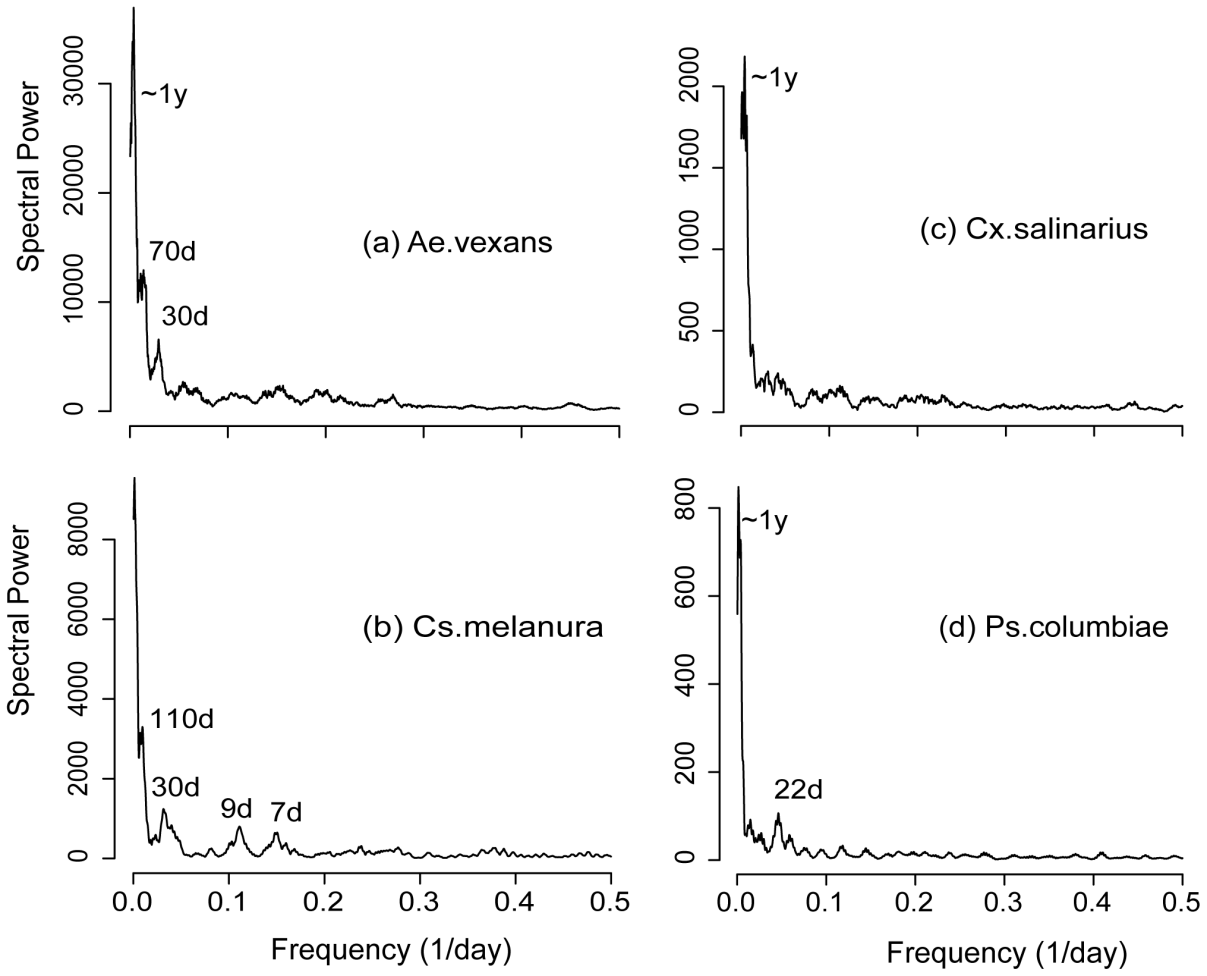
Power spectra for the 4 dominant species. The text marks the approximate locations of major peaks.

The results of DFT analysis differ for the four species (Figure 4). A Peak at yearly time scales in the power spectrum is found for *Ae.vexans*, *Cx. salinarius* and *Ps. columbiae*, but it is less obvious for *Cs.melanura*. The lack of detected cycles at yearly scales for Cs.melanura may probably be attributed to the large differences in its inter-annual abundance (e.g. see the particularly low abundance during the 2007 drought), as well as to more widely varying overwintering times (Figure 3). At the scale of 1 to several months, major peaks can be seen for *two* of the studied species (70 days and 30 days for *Ae.vexans*, and 110 days and 30 days for *Cs.melanura*). This intermediate range of time scales includes realistic durations of the life stages in mosquito life cycle [2], [3]. At shorter time scales (less than 1 month), peaks in the Fourier spectra are found for *Cs.melanura* and *Ps.columbiae*. In particular, peaks at wavelenghts shorter than 2 weeks are identified for *Cs.melanura*. Spectral power at daily scales does not exhibit significant preferred scales of fluctuations. The power spectra of temperature, rainfall and relative humidity exhibit a major annual cycle (Figure S3 in File S1). The spectrum of rainfall also shows peaks at time scales of several days, while the power spectrum of groundwater is very smooth with no obvious cycles during the study period. Interestingly, the peaks in the power spectra of mosquito abundance at monthly scales are not matched by major peaks in the power spectra of weather focings. This suggests that fluctuations over these time scales are caused by internal dynamical mechanisms.

**Figure 5:**
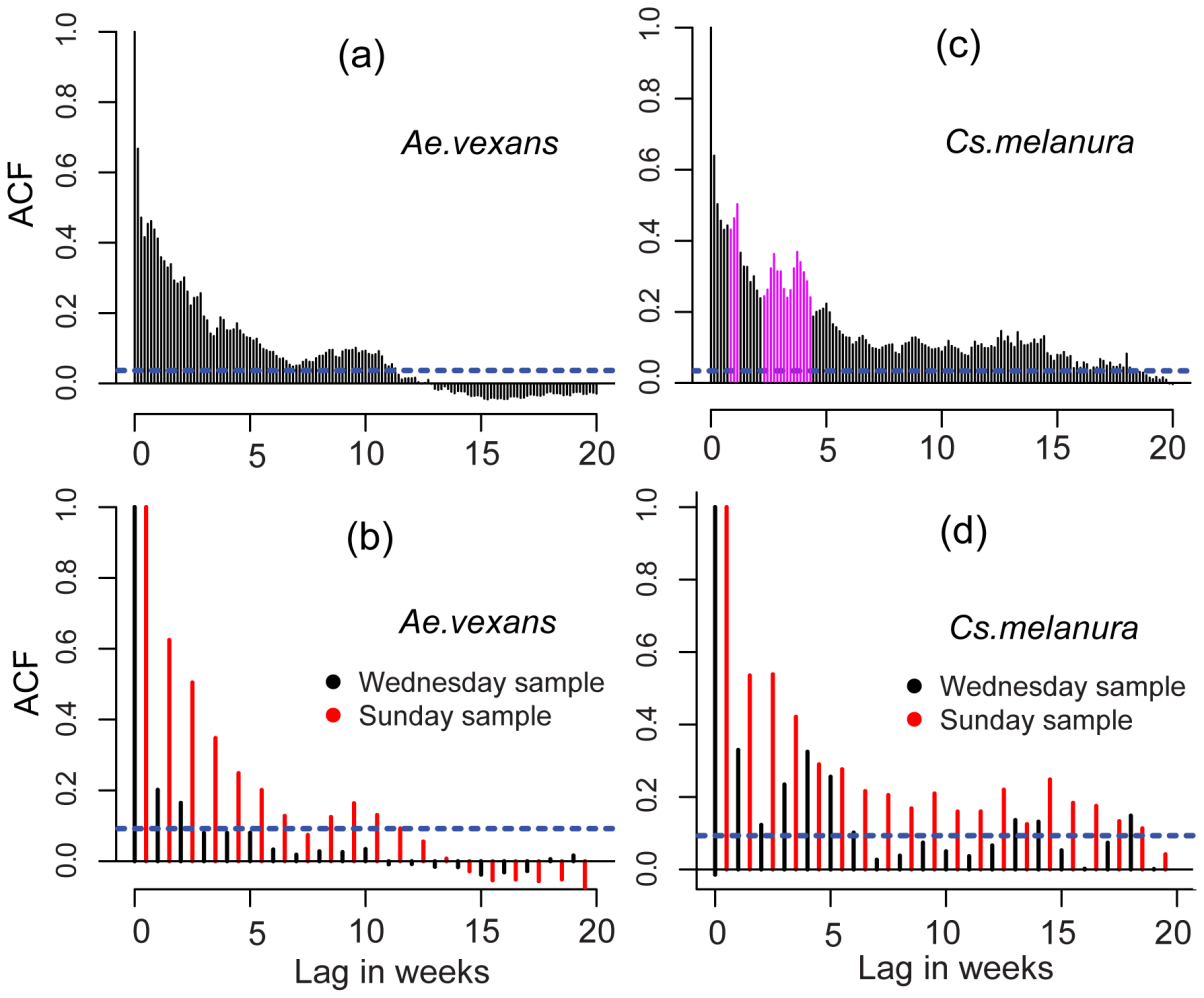
ACF for daily observations ((a) and (c)) and for subsampled weekly data ((b) and (d)). The dashed blue lines represent the 95% confidence intervals for the ACF. Significant peaks around 1 and 3–4 weeks, marked magenta in (c), are partly lost in the subsampled weekly data.

Analysis of the ACF's highlights significant differences between the daily data and the weekly subsamples, and among the weekly subsamples (Figure 5). For brevity we focus here, and in most of the following, on the two most relevant species, *Ae.vexans* and *Cs.melanura.* The ACFs of the daily *Ae.vexans* observations are significantly positive for time lags up to about 11 weeks, while, for example, the “Wednesday” samples for the same species only show a significant positive autocorrelation for lags up to 3 weeks. Furthermore, the ACFs of different weekly subsamples exhibit very wide differences. For example, the positive autocorrelations of the “Sunday” sample are both larger and more persistent than those in the “Wednesday” sample. A quite similar situation is seen in the case of *Cs.melanura*, some weekly samples suggesting a much shorter memory than others (Figure 5 c) and d)) and, in particular, a shorter memory than indicated by the more statistically representative daily observations. Furthermore, the peaks in the ACF of daily sampled *Cs.melanura* at about 1 and 3–4 weeks (Figure5, panel c) correspond to the peaks identified in the Fourier spectrum, as theory requires. Such peaks are lost in the weekly ACF. It is clear from these results that the apparent statistical properties of the adult mosquito population are extremely different depending on the specific weekly time series which happens to be sampled.

**Figure 6:**
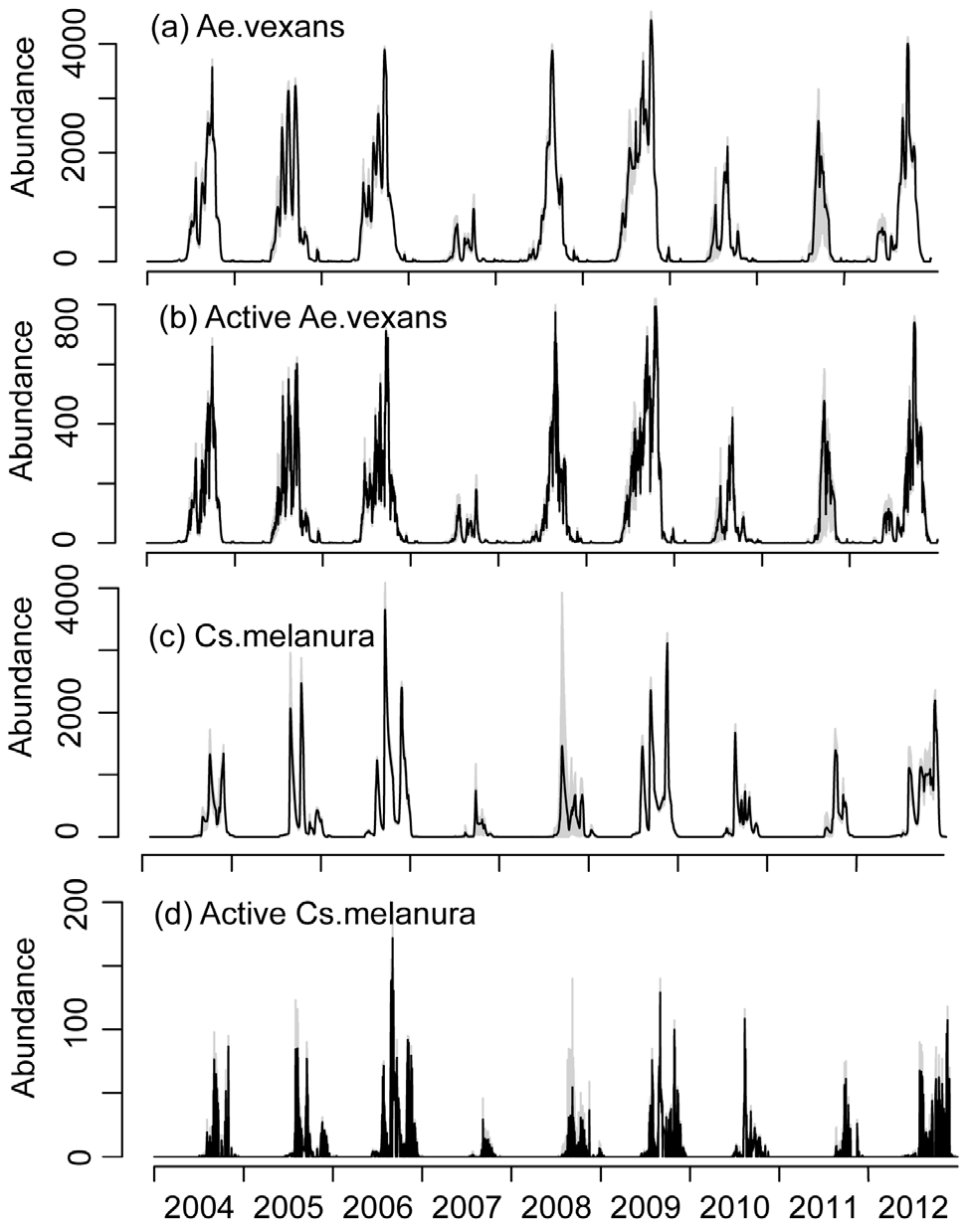
The mean abundance from 20 runs of IBS model driven by observed weather forcings for *Ae.vexans* and *Cs.melanura*: simulations without ((a) and (c)) and with ((b) and (d)) activity component. The grey lines represent the range (minimum and maximum) of 20 simulated values.

We now turn to the analysis of the synthetic time series generated using the IBS models. We again underline that the IBS models were run in an “unconstrained mode”: we used literature values of the parameters and we did not update the model state (number of eggs, adults, etc.) using observations during the simulations. The time series generated by the IBS models with observed environmental forcings with and without the activity term exhibit realistic annual peaks and reproduce the influence of dry and cold weather conditions (Figure 6). However, the fast time scales of fluctuation in the observed abundance are realistically represented only by the IBS model which includes an activity term modelling the variable vagility of adult mosquitoes (Figure 6).

**Figure 7:**
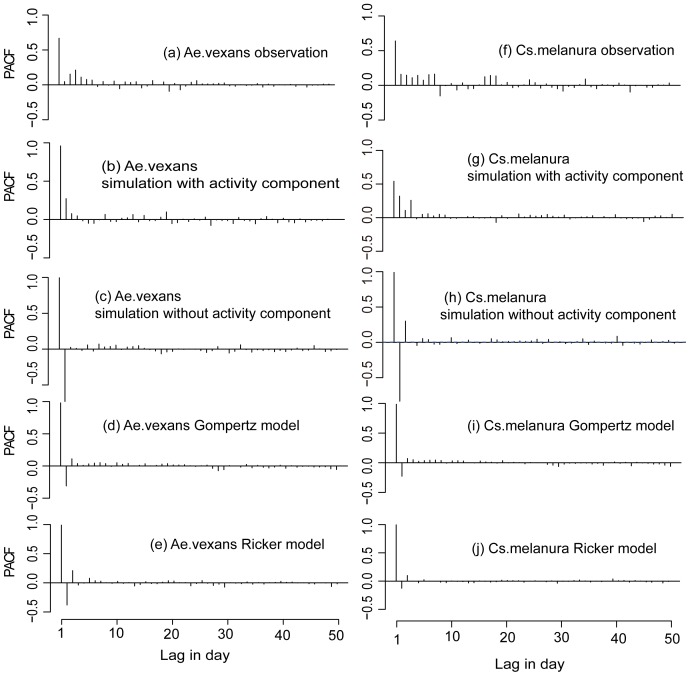
PACF of observed abundances for *Ae.vexans* and *Cs.melanura*, ((a) and (f)), for IBS model realizations including activity ((b) and (g)), IBS model realizations without activity ((c) and (h)); Gompertz model realizations with density dependence at lag = 0 days ((d) and (i)), and Ricker model realizations with density dependence at lag = 0 days [58] and (j)).

We also compared the PACFs of the observations, of the IBS simulations, and of the output of canonical population models. The PACFs for the daily observations are positive for both species up to about one week, while the PACFs generated by the Ricker and Gompertz models with density dependence dictated by the current abundance (lag 0) are negative at the same temporal scale (Figure 7 (d), (e), (i), (j)). Models which embed density dependence at the 1 day or 5-day lag exhibit similar results (Figure S4 in File S1). Moreover, adding multiple lags (lags 0–5) to the density dependence representation does not produce a better match of the observed correlations, as it increases the short term correlations but model outputs are less correlated in time than measured abundance (Figure S4 in File S1).

The PACFs for the IBS model without the activity component also show negative correlations at short temporal scale (Figure 7, (c) and (h)). However, when the activity component is included in the formulation, the resulting dynamics show significant, and realistic, positive correlations at time scales up to about one week (Figure 7, (b) and (g)). This suggests that the apparent correlation properties in the observed population at short time scales (<1 week) are highly influenced by adult mosquito activity and do not reflect fluctuations in the actual population.

**Figure 8:**
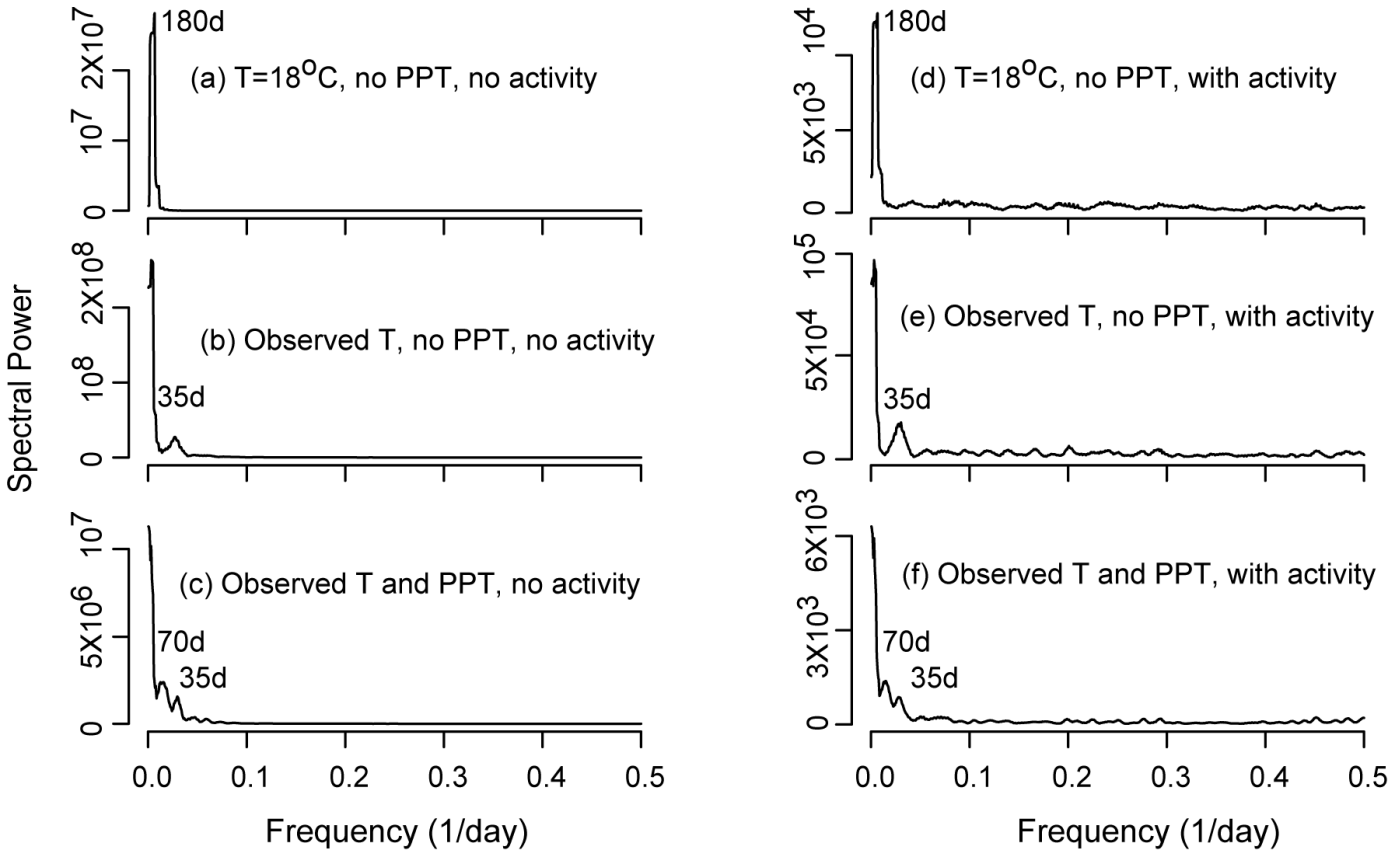
Power spectrum of IBS model outputs with ((a), (b), and (c)) and without ((d), (e), and (f)) activity component. Panel (a) and (d): IBS model with constant temperature (18°C) and no rainfall effect; panel (b) and (e); IBS model with observed temperature, and no rainfall effect; panel (c) and (f) IBS model with observed temperature, and with rainfall effect on death rate, oviposition, and egg hatching driven by observed rainfall.

We finally compare the power spectra of the simulated abundance generated with the three model configurations that include the activity component with those obtained by “turning-off” mosquito activity (Figure 8). For simplicity we use *Cs. melanura* as the illustrative example. Under constant environmental forcings, a major peak is found at time scales of about 180 days (Figure 8 (a) and (d)), which is a result of endogenous controls as exogenous factors do not fluctuate under this environmental setting (T = 18 °C, and no rainfall effect), with the population oscillating around the carrying capacity. When the observed temperature is included in the model, an annual peak appears due to the yearly cycle in this forcing (Figure 8 (b) and (e)). Under the same setting, the spectra also exhibit another peak at a monthly scale (about 35 days), which is the joint result of interacting endogenous and exogenous controls. The added rainfall effects reduce the size of the peaks at the yearly scale and introduce a peak at about 70 days (Figure 8 (c) and (f)). The annual cycle in the population abundance becomes less obvious because the interannual rainfall variability induces large uncorrelated variations in bloom times and abundance across different years. Finally, when activity is added to the model, the height of the peaks at longer time scales (>1 month) are reduced considerably while their position and shape are preserved. The amplitude of short time scale fluctuations increases (at the high-frequency end) relative to longer time scale periodicities, yielding a relatively low difference in amplitude across scales. While this is a qualitative observation, we note that model formulations including adult mosquito activity lead to power spectra in which energy decreases less steeply with increasing frequency, similarly to what happens for observations.

## Discussion and Conclusions

A coherent picture of adult mosquito dynamics, for the species examined here, emerges from the analysis of daily data and the use of a variety of modeling tools.

First, the analysis of the autocorrelation properties suggest that the relatively large “memory” in the observations (represented by positive values in the PACF for several time lags, Figure 7) can hardly be reproduced by density dependence mechanisms, even when multiple lags are involved. On the contrary, the inclusion of weather-driven activity does produce a modelled PACF which more realistically reproduces observed partial autocorrelations (see the supplementary material for a detailed comparison). The influence of mosquito activity on the ACF and PACF has implications for empirical analyses of mosquito populations. Observational ACFs and PACFs, in fact, importantly depend on the sampled abundance (as influenced by activity) and their interpretation as representative of the “memory” in the actual underlying population abundance can lead to erroneous conclusions regarding population dynamical mechanisms, e.g. in terms of reproductive time scales or of the time lags at which density dependence operates. The availability of daily observations allowed us to show that autocorrelation structures estimated on weekly data may vary widely and may be quite different from the “actual” autocorrelation structure estimated using daily data (Figure 5). Hence, even when relatively long time scales are of interest, a high sampling frequency is highly beneficial to obtain realistic estimates of abundance autocorrelation properties and of density dependent population regulation mechanisms.

**Figure 9:**
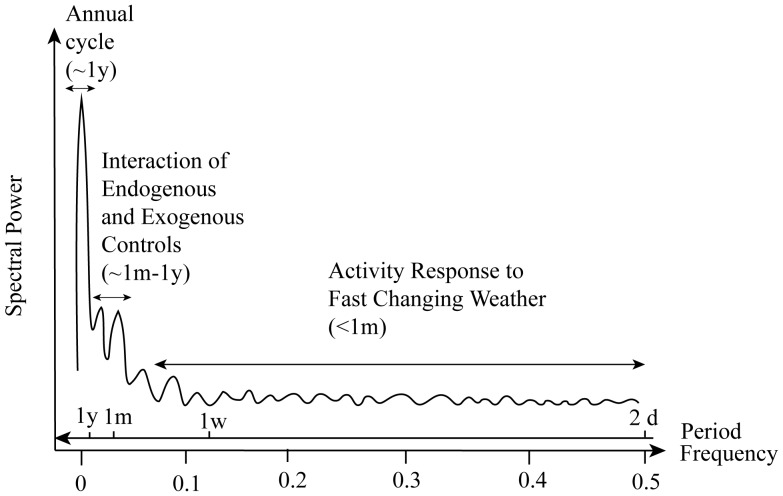
A conceptual spectrum highlighting the ecological/environmental processes driving mosquito dynamics at different time scales.

Spectral analysis reveals a coherent structure of the temporal organization of adult mosquito population dynamics (Figure 9). We find that mosquito activity, forced by short time scale weather, is most likely responsible for the observed population variability at fine time scales (<1 month). This is a sort of “microscale” at which variability is “injected” into the system. At monthly time scales coherent temporal structures and characteristic time scales emerge. These periodicities are here explained by the interplay of exogenous forcing and endogenous mechanisms. Our results suggest that the organized character of mosquito dynamics at the intermediate scales ranging from one to several months is jointly determined by the characteristic time scales of endogenous regulations (of survival, death rate, development, and reproduction) and by population responses to temperature and rainfall fluctuations At even longer time scales, mosquito population fluctuations mirror seasonal, annual, and inter-annual environmental patterns.

This interpretative framework provides guidance in choosing the observational temporal scale required to resolve the relevant population dynamics and possibly improve the prediction of adult mosquito population abundance. Clearly, if the mosquito species of interest exhibits characteristic scales of variability shorter than 2 weeks (such as in the case of *Cs.melanura*), our results imply that weekly observations, typical in adult mosquito studies in the field, are unsuitable to describe the full extent of the mosquito population variability, as they can only capture periodicities longer than two weeks.

Generation times in mosquito populations typically range between weeks and months [2], [3] and are thus not consistent with the rapid fluctuations we have observed in mosquito abundance on daily time scales. This discrepancy emphasizes the importance of understanding the relation between captured adult individuals and the actual underlying population. Non-detection by a trap does not imply the absence of the targeted animal [25], [51], and the number of captured individuals does not necessarily reflect true abundance fluctuations [24], [25], [52]–[56]. Hence, adult mosquito activity is a fundamental property of mosquito populations and fundamentally affects the observation process. Observations with weekly (or lower) resolution tend to overestimate population variability/responses because large observational fluctuations due to adult mosquito activity are erroneously attributed to changes in the actual population. Furthermore, we find that the inclusion of a weather-forced activity component allows the reproduction of realistic density dependence, autocorrelation functions, and power spectra.

Our results indicate that the dynamics of mosquito populations may not be understood separately from the mechanisms driving the activity of individuals. Current models for adult mosquitoes often appear unable to generate seemingly fast abundance fluctuations, and have difficulties in predicting observed per-capita growth rates at relatively short temporal scales [42], [57]. We suggest that this apparent lack of predictive ability may be due to a changing proportion of the active mosquito individuals, which should be included as a fundamental ingredient of mosquito population models, conceptual or mechanistic, particularly at the short temporal scales.

## Supporting Information

File S1 **Online supporting figures.**
(PDF)

File S2 **Detailed functions and parameter values used in the IBS models.**
(DOC)

File S3 **Estimated coefficients for density dependent models.**
(DOC)

File S4 **Readme file for the data.**
(DOC)

File S5 **Mosquito abundance data.**
(CSV)
