# Supplementary material for: The Temporal Spectrum of Adult Mosquito Population Fluctuations: Conceptual and Modeling Implications
Source: PLoS One. 2014 Dec 5;9(12):e114301. doi: 10.1371/journal.pone.0114301 (PMC4257610; doi:10.1371/journal.pone.0114301)
Supplement: File S1 — Online supporting figures. (PDF) [file pone.0114301.s001.pdf]

**Supporting figures**

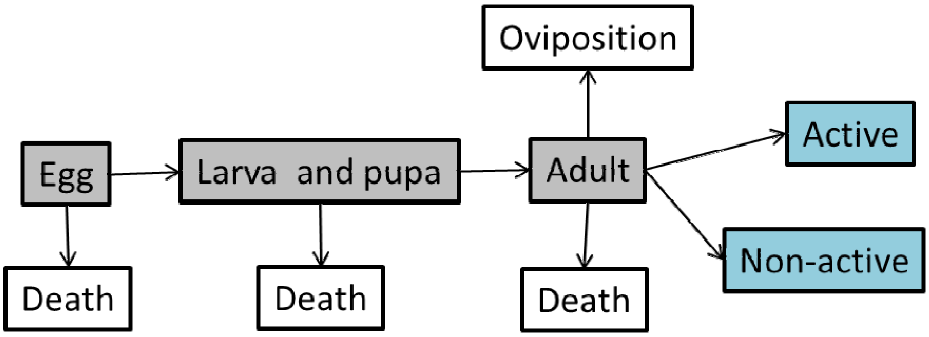


Figure S1. IBS model formulation, developmental stages, and processes.

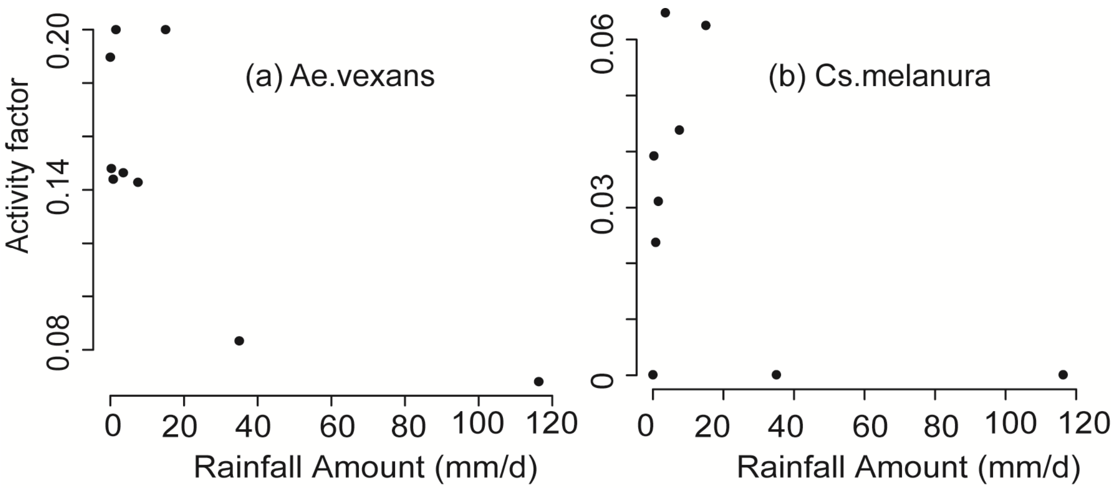


Figure S2. Empirical relationship between activity factor and rainfall

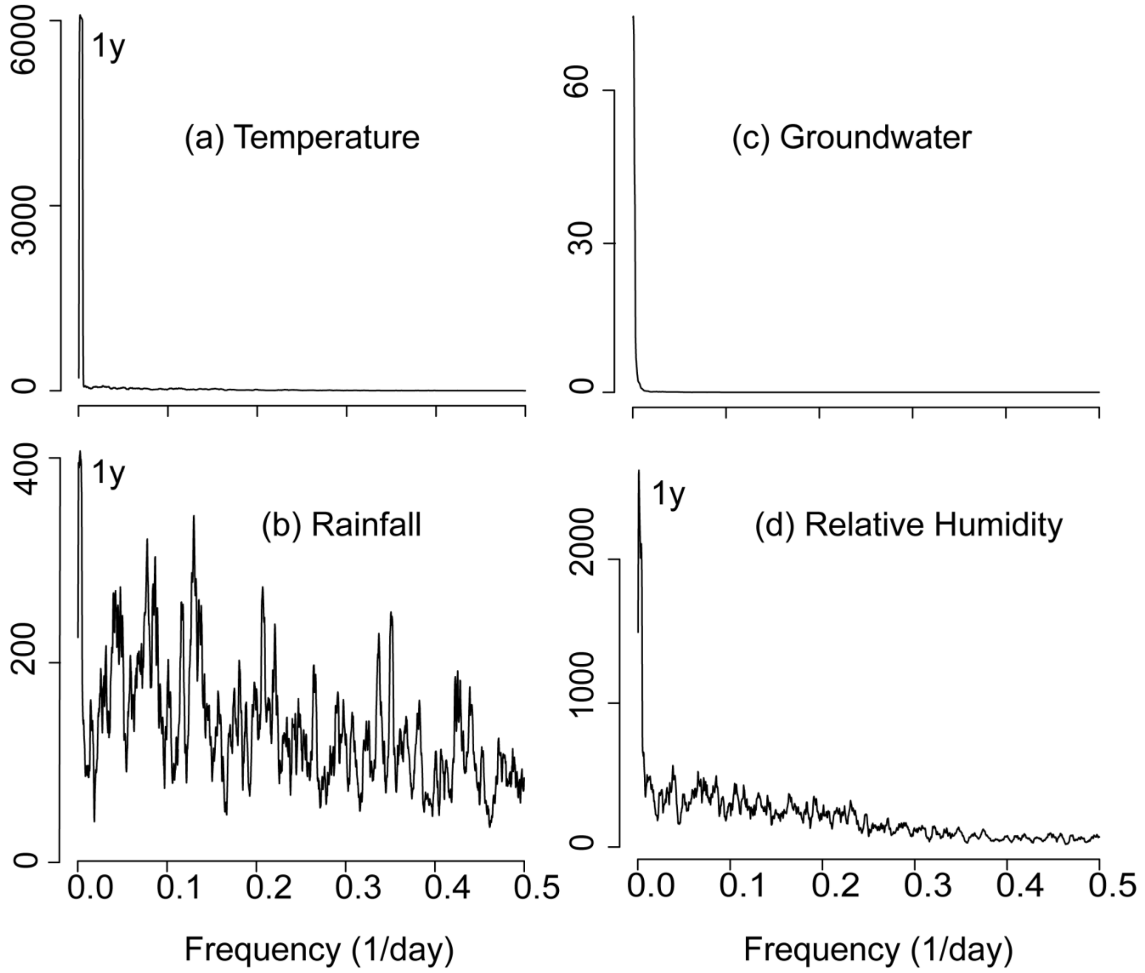


Figure S3: Power spectrum for the weather observations.

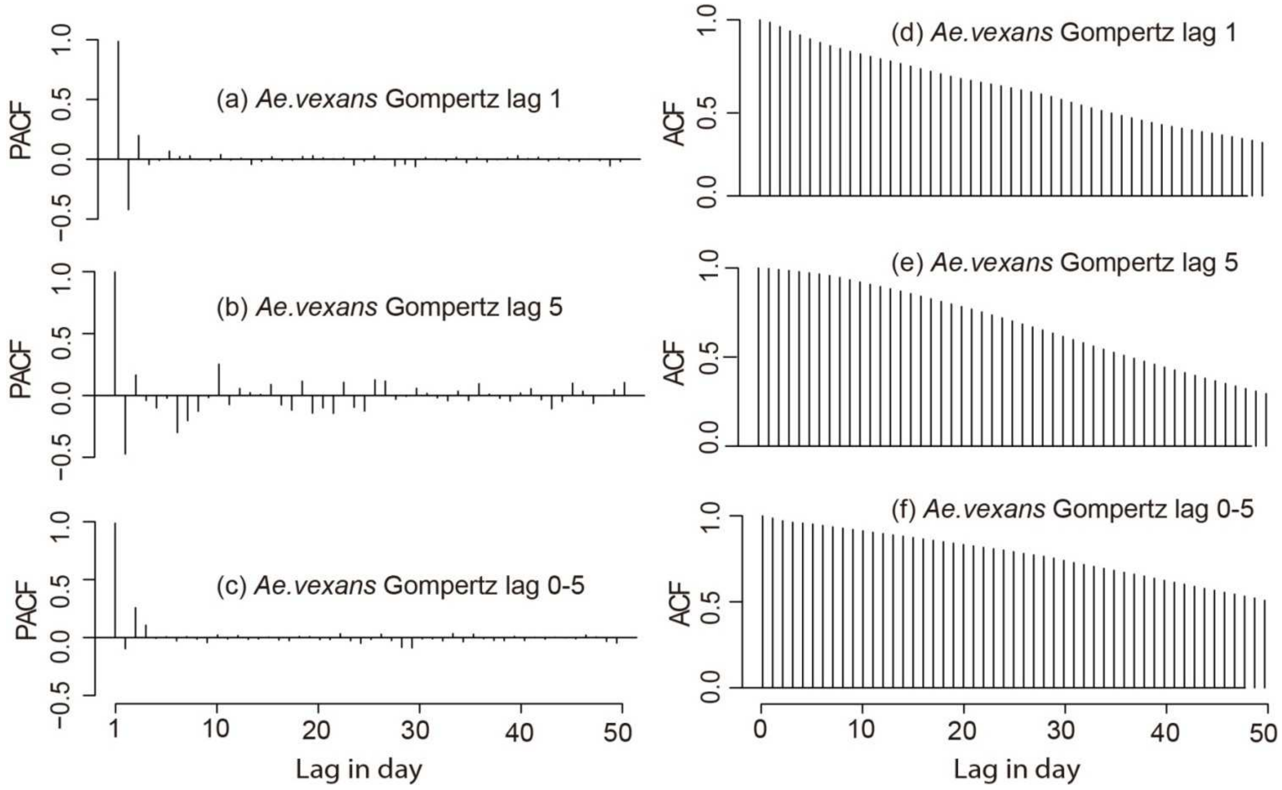


Figure S4 ACF and PACF of Gompertz models for *Ae.vexans* with different density dependence (results for the Ricker model, not shown, are analogous)

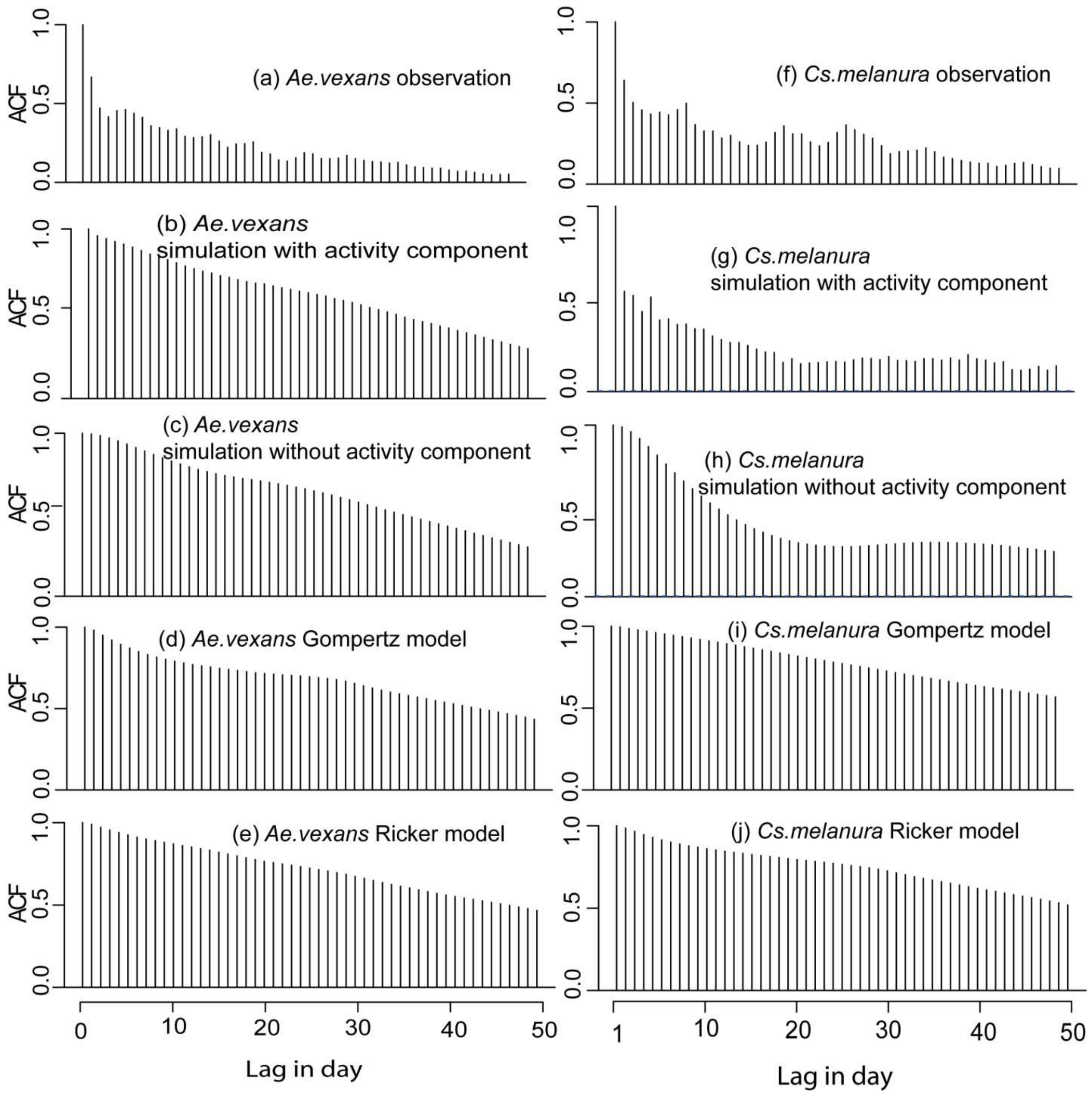


Figure S5. ACF for observations and for models with different density dependence. ACF of observed abundances for *Ae.vexans* and *Cs.melanura* ((a) and (f)), for IBS model realizations including activity ((b) and (g)), IBS model realizations without activity ((c) and (h)) ; Gompertz model realizations with density dependence at lag=0 days ((d) and (i)) , and Ricker model realizations with density dependence at lag=0 [1] and (j)).
