# Supplementary material for: The Temporal Spectrum of Adult Mosquito Population Fluctuations: Conceptual and Modeling Implications
Source: PLoS One. 2014 Dec 5;9(12):e114301. doi: 10.1371/journal.pone.0114301 (PMC4257610; doi:10.1371/journal.pone.0114301)
Supplement: File S2 — Detailed functions and parameter values used in the IBS models. (DOC) [file pone.0114301.s002.doc]

**S2. Detailed functions and parameter values used in the IBS models**

The IBS models explicitly describe three mosquito life stages: egg, larva/pupa, and adult . Each stage is characterized by a distribution of the time spent in that stage, dependent on physiology, environmental conditions, and population density. Each stage is characterized by a survival rate, also a function of environmental conditions. We based our description of the distributions of the time spent in each stage and of the survival rate for each stage on existing literature, to minimize the number of parameters which require ad hoc calibration (Figure 2).

For *Ae.vexans*, when the 10 days moving-average of daily temperature (temp10) is between 10-30^o^C, a proportion of these eggs hatch . The state of an egg is drawn from a Bernoulli distribution, where 1 represents a hatched egg while 0 represents unhatched one. Because rainfall can increase oviposition sites, we further assume that the hatching portion increases linearly with the 10 days moving-average of rainfall (ppt10, *p* = 0.01+*ppt10*×0.015 in the Bernoulli distribution). The expected number of days needed to develop from eggs to the 1^st^ instar of larva is assumed to decrease with the temperature according to a power law (1808× *temp10^-^*^1.90^ ) . The actual length of development is drawn from a normal distribution with the mean mentioned above and a standard deviation of 1 day. Because the eggs of *Ae.vexans* can live for years, we assume the daily survival rate for the eggs is high and decrease with the number of eggs (1- 0.00002 × *Negg*) . In the pupal/larval stage, the assumed mean development time is 2981× *temp10^-^*^1.86^+4 . The actual length of development is drawn from a normal distribution with the this mean and a standard deviation of 1 day. The daily survival rate for the pupal/larval stage is assumed to be a quadratic function of temperature and decreases with the number of larvae because of potential competition for nutrients 0.9173 + 0.0060 × *temp10* - 0.00015 × *temp10^2^* – 0.00002 ×*Nlarva*. Furthermore, we assumed this rate decreases by 20% when ppt10 is smaller than 1mm/d due to drying out of the habitats, and decrease by 50% when temp10 is smaller than 10^o^C. After adult emergence, a proportion of the females are successful in finding blood meal (Bernoulli (*p* = 0.8+ *RH10*/100) for *Ae.vexans*, where RH10 is the 10 days moving-average of relative humidity). The length of gonotrophic cycle is assumed to be drawn from a truncated normal distribution with the mean equals to 10 days, standard deviation equals to 1day, the minimum length equals to 7 days, and the maximum length equals to 13 days . The number of eggs laid by a female per batch is a random number from a normal distribution with the mean equals to 100+ppt10, the standard deviation equals to 10, and the lower bound equal to 0. The daily survival rate of adult females is assumed to be a quadratic function of temperature ( 0.5 + 0.048 × *temp10* -0.0012 × *temp10^2^*). Furthermore, we assumed this rate decreases by 20% when ppt10 is smaller than 1mm/d, and decreases by 50% when temp10 is smaller than 10^o^C. Finally, the proportion of the active mosquitoes is calculated based on the observed data. We first calculated the ratio of the current abundance divided by the maximum abundance for moving window of 31 days, using the maximum abundance as an approximation of the true population. This ratio is then plotted against current day rainfall, which we assumed to be the main driver of the activity factor (see online supporting material). This plot suggested a nonlinear relationship between the ratio and the current day rainfall (Figure S4). The median of the ratio for each rainfall range is used as the activity factor.

The simulation setup for *Cs.melanura* is similar. Hence we only describe the differences in the model formulations for the two species. The temperature limits for egg hatching are 5 to 32^o^C . The expected number of days needed to develop from eggs to the 1^st^ instar of larva is 4230× *temp10^-^*^2.30^ . In the pupal/larval stage, the assumed mean development time is 43478× *temp10^-^*^2.25^. The daily egg survival rate is assumed to be 1-0.000004*Negg. The daily survival rate for pupal/larval stage is assumed to be 1- 0.000004× *Nlarva*, and if ppt10 is smaller than 1 mm/d, the value is decreased by 20%. The proportion of females succeeding in finding a blood meal and ovipositing is 0.419 +0.133×*temp10*-0.0035× *temp10*^2^ when 7.5 ≤ *temp10* ≤28.5^o^C . The daily survival rate of adult females is assumed to be a quadratic function of temperature (0.5 + 0.048 × *temp10* -0.0012 × *temp10^2^*), and it is decreased by 20% when ppt10 is smaller than 1mm/d. The expected length of the gonotrophic cycle is 95.87/(*temp10* -6.4) . The number of eggs laid per batch is from a normal distribution with the mean equals to 93+ppt10, the standard deviation equals to 34, and the lower bound equal to 0 .

**Reference**
