# Supplementary material for: The Temporal Spectrum of Adult Mosquito Population Fluctuations: Conceptual and Modeling Implications
Source: PLoS One. 2014 Dec 5;9(12):e114301. doi: 10.1371/journal.pone.0114301 (PMC4257610; doi:10.1371/journal.pone.0114301)
Supplement: File S3 — Estimated coefficients for density dependent models. (DOC) [file pone.0114301.s003.doc]

**S3**. Estimated coefficients for density dependent models

| ***Cs.melanura*** | | ***Ae.vexans*** | |
| --- | --- | --- | --- |
| **Gompertz lag0** | **Estimate** | **Gompertz lag0** | **Estimate** |
| Intercept | 0.145** | Intercept | 0.294*** |
| daily_logN | -0.166*** | daily_logN | -0.165*** |
| daily_temp | 0.005 | daily_temp | 0.022** |
| daily_temp2 | 0.000 | daily_temp2 | 0.000* |
| daily_ppt | 0.001 | daily_ppt | -0.001 |
| daily_RH | -0.002** | daily_RH | -0.005*** |
| **Gompertz lag1** | **Estimate** | **Gompertz lag1** | **Estimate** |
| Intercept | 0.179** | Intercept | 0.318*** |
| daily_logN1 | -0.061*** | daily_logN1 | -0.073*** |
| daily_temp | -0.002 | daily_temp | 0.010 |
| daily_temp2 | 0.000 | daily_temp2 | 0.000 |
| daily_ppt | 0.001 | daily_ppt | -0.001 |
| daily_RH | -0.002** | daily_RH | -0.004*** |
| **Gompertz lag5** | **Estimate** | **Gompertz lag5** | **Estimate** |
| Intercept | 0.205*** | Intercept | 0.373*** |
| daily_logN5 | -0.007 | daily_logN5 | -0.005 |
| daily_temp | -0.005 | daily_temp | 0.002 |
| daily_temp2 | 0.000 | daily_temp2 | 0.000 |
| daily_ppt | 0.001 | daily_ppt | -0.001 |
| daily_RH | -0.002** | daily_RH | -0.005*** |
| **Gompertz lag0 to lag5** | **Estimate** | **Gompertz lag0 to lag5** | **Estimate** |
| Intercept | 0.104* | Intercept | 0.280*** |
| daily_logN | -0.500*** | daily_logN | -0.405 |
| daily_logN1 | 0.051** | daily_logN1 | 0.033 |
| daily_logN2 | 0.107*** | daily_logN2 | 0.065** |
| daily_logN3 | 0.086*** | daily_logN3 | 0.109*** |
| daily_logN4 | 0.108*** | daily_logN4 | 0.053** |
| daily_logN5 | 0.085*** | daily_logN5 | 0.063*** |
| daily_temp | 0.008 | daily_temp | 0.021** |
| daily_temp2 | 0.000 | daily_temp2 | 0.000* |
| daily_ppt | 0.000 | daily_ppt | -0.002* |
| daily_RH | -0.002** | daily_RH | -0.005*** |
| **Ricker lag0** | **Estimate** | **Ricker lag0** | **Estimate** |
| Intercept | 0.171** | Intercept | 0.359*** |
| daily_N | -0.006*** | daily_N | -0.004*** |
| daily_temp | -0.002 | daily_temp | 0.008 |
| daily_temp2 | 0.000 | daily_temp2 | 0.000 |
| daily_ppt | 0.001 | daily_ppt | -0.001 |
| daily_RH | -0.002** | daily_RH | -0.005*** |
| **Ricker lag1** | **Estimate** | **Ricker lag1** | **Estimate** |
| Intercept | 0.184** | Intercept | 0.343*** |
| daily_N1 | -0.004*** | daily_N1 | -0.003*** |
| daily_temp | -0.003 | daily_temp | 0.006 |
| daily_temp2 | 0.000 | daily_temp2 | 0.000 |
| daily_ppt | 0.001 | daily_ppt | -0.001 |
| daily_RH | -0.002** | daily_RH | -0.004*** |
| **Ricker lag5** | **Estimate** | **Ricker lag5** | **Estimate** |
| Intercept | 0.205*** | Intercept | 0.373*** |
| daily_N5 | 0.000 | daily_N5 | 0.000 |
| daily_temp | -0.006 | daily_temp | 0.002 |
| daily_temp2 | 0.000 | daily_temp2 | 0.000 |
| daily_ppt | 0.001 | daily_ppt | -0.001 |
| daily_RH | -0.002** | daily_RH | -0.005*** |
| **Ricker lag0 to lag5** | **Estimate** | **Ricker lag0 to lag5** | **Estimate** |
| Intercept | 0.151** | Intercept | 0.365*** |
| daily_N | -0.008*** | daily_N | -0.004*** |
| daily_N1 | -0.003** | daily_N1 | -0.002** |
| daily_N2 | 0.002* | daily_N2 | 0.000 |
| daily_N3 | 0.002. | daily_N3 | 0.003*** |
| daily_N4 | 0.002 | daily_N4 | -0.001 |
| daily_N5 | 0.001 | daily_N5 | 0.002** |
| daily_temp | -0.003 | daily_temp | 0.004 |
| daily_temp2 | 0.000 | daily_temp2 | 0.000 |
| daily_ppt | 0.000 | daily_ppt | -0.001 |
| daily_RH | -0.002* | daily_RH | -0.005*** |

levels of significance are: * p <0.1, ** p<0.05, and *** p<0.01
