# Supplementary material for: The Temporal Spectrum of Adult Mosquito Population Fluctuations: Conceptual and Modeling Implications
Source: PLoS One. 2014 Dec 5;9(12):e114301. doi: 10.1371/journal.pone.0114301 (PMC4257610; doi:10.1371/journal.pone.0114301)
Supplement: File S4 — Readme file for the data. (DOC) [file pone.0114301.s004.doc]

The dataset ‘Brunswick Mosquitoes.csv’ contains daily mosquito samples collected from 2004 to 2013 in the Brunswick County North Carolina, USA. The mosquito samples were collected using New Jersey light traps at 3 sites “Chix”, “Fox”, and “Xroads”. The locations of the sites are listed below. The light traps collected the mosquito samples all year around continuously. These traps were maintained by the Mosquito Control department, Brunswick County Government.

| **Directions** | **Physical Address** | **Habitat** | **Datum WGS 84?** | **GPS Coordinates North** | **GPS Coordinates West** |
| --- | --- | --- | --- | --- | --- |
| Chicken Trap | 179 March 9, 1764 | Woodland Pool | Yes | 34.05883333 | -78.16756667 |
| Fox Trap | 522 Grayfox Court | Urban | Yes | 34.21577000 | -77.99665000 |
| X-Roads | 1159 Hickman Rd | Rural Container | Yes | 33.92962000 | -78.61124000 |
